# Approaches to Gene Mutation Analysis Using Formalin-Fixed Paraffin-Embedded Adrenal Tumor Tissue From Patients With Primary Aldosteronism

**DOI:** 10.3389/fendo.2021.683588

**Published:** 2021-06-29

**Authors:** Kazutaka Nanba, William E. Rainey, Aaron M. Udager

**Affiliations:** ^1^ Department of Molecular and Integrative Physiology, University of Michigan, Ann Arbor, MI, United States; ^2^ Department of Endocrinology and Metabolism, National Hospital Organization Kyoto Medical Center, Kyoto, Japan; ^3^ Division of Metabolism, Endocrinology, and Diabetes, Department of Internal Medicine, University of Michigan, Ann Arbor, MI, United States; ^4^ Department of Pathology, University of Michigan, Ann Arbor, MI, United States; ^5^ Rogel Cancer Center, University of Michigan, Ann Arbor, MI, United States; ^6^ Michigan Center for Translational Pathology, University of Michigan, Ann Arbor, MI, United States

**Keywords:** primary aldosteronism, CYP11B2, somatic mutation, immunohistochemistry, next-generation sequencing

## Abstract

Aldosterone production is physiologically under the control of circulating potassium and angiotensin II as well as adrenocorticotropic hormone and other secretagogues such as serotonin. The adrenal’s capacity to produce aldosterone relies heavily on the expression of a single enzyme, aldosterone synthase (CYP11B2). This enzyme carries out the final reactions in the synthesis of aldosterone and is expressed almost solely in the adrenal zona glomerulosa. From a disease standpoint, primary aldosteronism (PA) is the most common of all adrenal disorders. PA results from renin-independent adrenal expression of CYP11B2 and production of aldosterone. The major causes of PA are adrenal aldosterone-producing adenomas (APA) and adrenal idiopathic hyperaldosteronism. Our understanding of the genetic causes of APA has significantly improved through comprehensive genetic profiling with next-generation sequencing. Whole-exome sequencing has led to the discovery of mutations in six genes that cause renin-independent aldosterone production and thus PA. To facilitate broad-based prospective and retrospective studies of APA, recent technologic advancements have allowed the determination of tumor mutation status using formalin-fixed paraffin-embedded (FFPE) tissue sections. This approach has the advantages of providing ready access to archival samples and allowing CYP11B2 immunohistochemistry-guided capture of the exact tissue responsible for inappropriate aldosterone synthesis. Herein we review the methods and approaches that facilitate the use of adrenal FFPE material for DNA capture, sequencing, and mutation determination.

## Introduction

Technologic advances in genetic analysis have provided us a better understanding of the molecular pathogenesis of endocrine-related tumors. Aldosterone-producing adenoma (APA) is one of the major subtypes of primary aldosteronism (PA), the most common cause of endocrine-related hypertension. The application of next-generation sequencing (NGS) has resulted in the identification of disease-causing mutations in APA and familial PA. Aldosterone-driver mutations can occur in genes encoding membrane ion channels or pumps (1–9). Thus far, APA have been found with mutations in *KCNJ5* (1)*, ATP1A1* (2, 4), *ATP2B3* (2), *CACNA1D* (3, 4), *CACNA1H* (9), and *CLCN2* (8) (aldosterone-driver mutations). These mutations cause excess aldosterone production by raising intracellular calcium levels which leads to enhanced CYP11B2 (aldosterone synthase) expression and renin-independent aldosterone production (10). Like other adrenocortical tumors, activating mutations in *CTNNB1* gene (encoding β-catenin) have also been documented in a subset of APA (11–14) but the mechanism of *CTNNB1* mutation activation of aldosterone production remains to be clearly defined. So far more than 90 APA somatic mutations have been reported (**Table 1**). Of note, only part of the previously reported somatic mutations has been functionally tested so far. To assess the pathologic role of these mutations, it would be ideal to perform cell-based studies for each mutation. In addition to tumor somatic mutations, PA aldosterone production may be regulated by hormones that include adrenocorticotropic hormone, serotonin, or luteinizing hormone (37–44).

**Table 1:** Previously reported somatic mutations in aldosterone-producing adenomas.

Gene	Somatic Mutations	
***KCNJ5***	c.343C>T (p.R115W) (15)	c.445_446insTGG (p.T149delinsMA) (49)
	c.376T>C (p.W126R) (16)	c.447_448insATT (p.T149delinsTI) (14)
	c.414_425dupGCTTTCCTGTTC (p.A139_F142dup) (17)	c.450_451insATG (p.I150_G151insM) (13)
	c.420C>G (p.F140L) (14)^a^	c.451G>A (p.G151R) (1)
	c.433_434insCCATTG (p.I144_E145insAI) (13)	c.451G>C (p.G151R) (24)
	c.433G>C (p.E145Q) (18)	(p.G151_Y152del)* (25)
	c.433G>A (p.E145K) (4)	c.457_492dupG_G (p.G153_G164dup) (20)
	c.432_439delTGAGACCGinsCA (p.E145_E147delinsK) (19)	c.461T>G (p.F154C) (13)
	c.439G>C and c.448_449insCAACAACCA (p.E147Q_T149_I150insTTT) (20)	c.467_469delTCA (p.I157del) (26)
	c.443C>T (p.T148I) (21)^b^	c.470_471delinsAA (p.I157K) (13)
	c.445_446insGAA (p.T148_T149insR) (22)	(p.I157_E159del)* (25)
	c.446insAAC (p.T149_I150insT) (23)	c.472A>G (p.T158A) (27)
	c.445A>T (p.T149S) (21)	c.503T>G (p.L168R) (1)
		(p.G184E)* (25)
		c.737A>G (p.E246G) (15)
***ATP1A1***	c.295G>A (p.G99R) (16)	c.2874_2882delCTTTGAAGA (p.F959_E961del) (29)
	c.299_313delTCTCAATGTTACTGT (p.F100_L104del) (2)	c.2877_2882delTGAAGA (p.F959_E961delinsL) (28)
	c.304_309delATGTTA (p.M102_L103del) (28)	c.2878_2895delGAAGAGACAGCCCTGGCTinsGCCCTGGTT (p.E960_A965delinsALV) (48)
	c.306_317delGTTACTGTGGAT (p.M102_I106delinsW) (28)	c.2877_2888delTGAAGAGACAGC (p.E960_A963del) (29)
	c.308_313delTACTGT (p.L103_L104del) (28)	c.2878_2887delGAAGAGACAGinsT (p.E960_A963delinsS) (4)
	c.311T>G (p.L104R) (2)	c.2879_2890delAAGAGACAGCCC (p.E960_L964delinsV) (28)
	c.995T>G (p.V332G) (2)	c.2878_2892delGAAGAGACAGCCCTGinsGCCGTG (p.E960_L964delinsAV) (14)
	c.2864_2878delTATTTGGCCTCTTTG (p.I955_E960delinsK) (49)	
	c. 2867_2882delTTGGCCTCTTTGAAGAinsG (p.F956_E961delinsW) (28)	
***ATP2B3***	c.367G>C (p.G123R) (30)	c.1273_1278delCTGGTC (p.L425_V426del) (2)
	c.1228T>G (p.Y410D) (31)	c.1277_1282delTCGTGG (p.V426_V427del) (2)
	c.1264_1278delGTCACTGTGCTGGTCinsAGCACACTC (p.V422_V426delinsSTL) (22)	c.1276_1287delGTCGTGGCTGTC (p.V426_V429del) (28)
	c.1264_1275delGTCACTGTGCTGinsATCACT (p.V422_L425delinsIT) (14)	c.1276_1298insGACA_delTCGTGGCTGTCCCAGAGGGCCT (p.V426G_V427Q_A428_L433del) (13)
	c.1269_1274delTGTGCT (p.V424_L425del) (32)	c.1279_1284delGTGGCT (p.V427_A428del) (33)
	c.1270_1275delGTGCTG (p.V424_L425del) (55)	c.1281_1286delGGCTGT (p.A428_V429del) (34)
	c.1272_1277delGCTGGT (p.L425_V426del) (2)	
***CACNA1D***	c.776T>A (p.V259D) (4)	c.2906C>T (p.S969L) (48)
	c.776T>G (p.V259G) (14)	c.2936T>A (p.V979D) (55)
	c.926T>C (p.V309A) (49)	c.2943G>C (p.V981N) (55)
	c.1201C>G (p.V401L) (28)	c.2968C>G (p.R990G) (49)
	c.1207G>C [p.G403R (exon8A)] (3, 4)	c.2969G>A (p.R990H) (4)
	c.1207G>C [p.G403R (exon8B)] (3)**	c.2978G>C (p.R993T) (49)
	c.1229C>T (p.S410L) (30)	c.2978G>T (p.R993M) (29)
	c.1856G>C (p.R619P) (49)	c.2992_2993GC>AT (p.A998I) (55)
	c.1955C>T (p.S652L) (55)	c.2993C>T (p.A998V) (55)
	c.1964T>C (p.L655P) (55)	c.3019T>C (p.C1007R) (49)
	c.2182G>A (p.V728I) (20)	c.3044T>G (p.I1015S) (49)
	c.2222A>G (p.Y741C) (55)	c.3044T>C (p.I1015T) (58)
	c.2239T>G (p.F747V) (3)	c.3451G>T (p.V1151F) (55)
	c.2239T>C (p.F747L) (4)	c.3452T>C (p.V1151A) (29)
	c.2241C>G (p.F747L) (4)	c.3455T>A (p.I1152N) (55)
	c.2240T>G (p.F747C) (56)	c.3458T>G (p.V1153G) (35)
	c.2240T>C (p.F747S) (29)^c^	c.4007C>G (p.P1336R) (4)
	c.2250C>G (p.I750M) (3, 4)	c.4012G>A (p.V1338M) (3)
	c.2248A>T (p.I750F) (55)	c.4062G>A (p.M1354I) (4)
	c.2261A>G (p.N754S) (29)	
***CACNA1H***	c.4289T>C (p.I1430T) (9)	
***CLCN2***	c.71G>A (p.G24D) (8)	
	c.64-2_74del (36)	

^a-c^Associated with another somatic mutation (^a^KCNJ5 p.G151R; ^b^KCNJ5 p.T149S; ^c^CACNA1D p.N754S). * Base change information was not provided in the original article. For the CACNA1D mutations, amino acid substitutions are described based on the reference sequence NM_001128839 otherwise noted (**NM_000720 for the mutation in exon 8B). Mutations that are covered by the primer sets in **Table 2** are highlighted in blue.

Since the development of specific antibodies against human CYP11B2, which is required for aldosterone biosynthesis, CYP11B2 immunohistochemistry (IHC) has played an important role in defining the histopathologic characteristics of adrenals from patients with PA (45, 46). CYP11B2 IHC has revealed diversities in the histopathology of adrenals from patients with PA, including APA (CYP11B2-expressing adrenocortical adenoma) and adrenals with small CYP11B2-expressing cell nests, called aldosterone-producing cell clusters (APCCs) (45) or aldosterone-producing micronodules (APMs) (47). Advanced sequencing methods combined with CYP11B2 IHC have significantly improved the detection rate of somatic mutations in APA (14, 48, 49). CYP11B2 IHC-guided targeted NGS has also allowed the detection of aldosterone-driver mutations in APCCs (APMs) using small amounts of DNA (50–52). Herein, we provide an overview of recent advances in the genetic analysis of APA and introduce a streamlined sequencing approach using formalin-fixed paraffin-embedded (FFPE) tumor tissue material.

## Importance of CYP11B2 IHC and Targeted DNA Capture

Development of specific antibodies against human CYP11B2 has allowed detection of the source of pathologic aldosterone production in the resected adrenal tissue (45, 46). Unique characteristics of adrenals from patient with PA have been documented by CYP11B2 IHC. Importantly, adrenal tumors detected by cross-sectional imaging study are not always the cause of aldosterone excess even when adrenal vein sampling lateralizes autonomous aldosterone production to the tumor side (53). In such cases, APA can be below the detection limit of imaging studies and/or imaging-detected tumors can be non-functioning adrenocortical adenomas (CYP11B2-negative tumors by IHC). Cases with multiple APAs within one adrenal have also been documented (14, 48, 49, 54).

**Figure 1:**
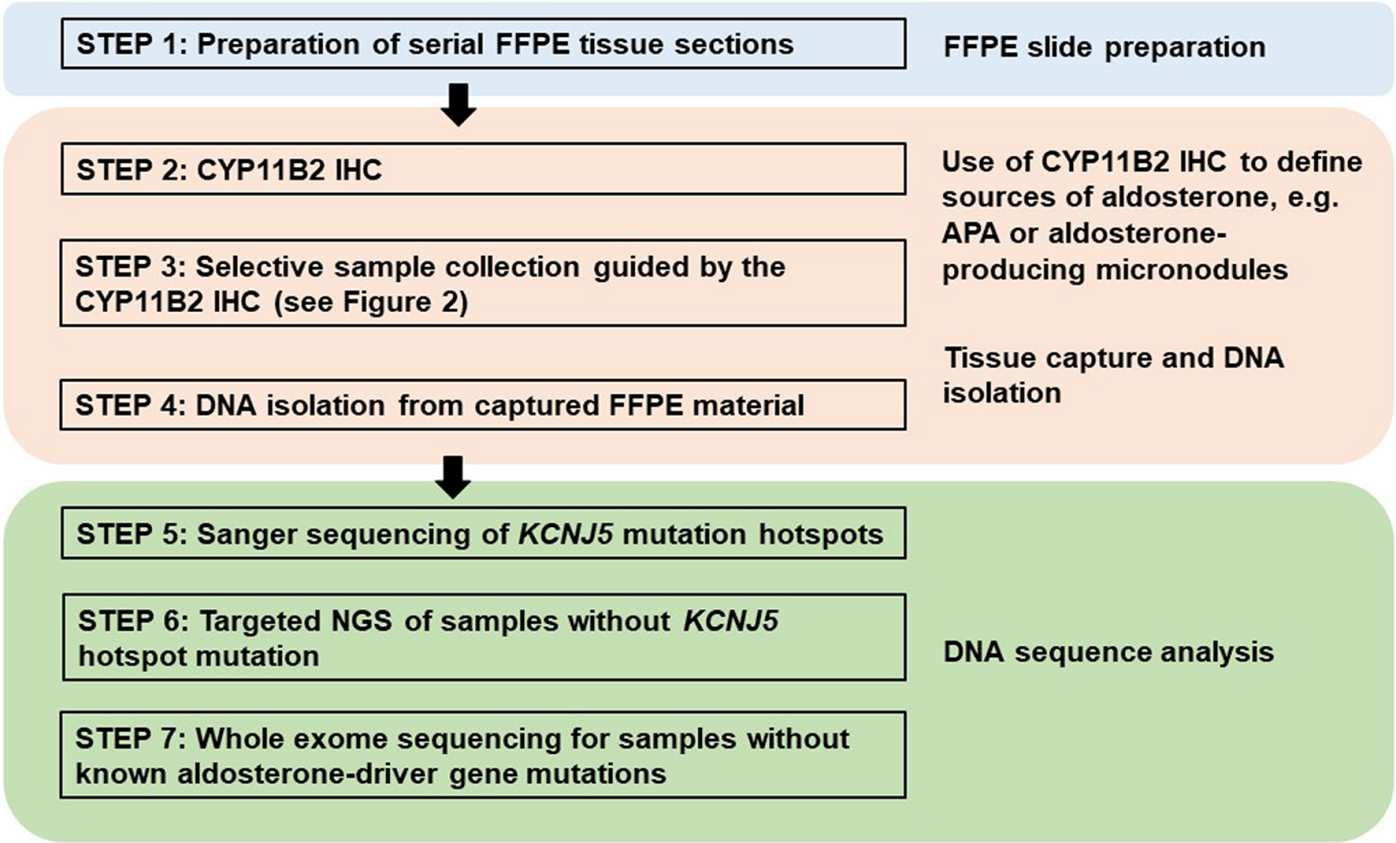
Proposed method for DNA mutation analysis using excised adrenal tissue sections from patients with primary aldosteronism. This approach uses CYP11B2 immunohistochemistry (IHC) to define the source of aldosterone for DNA capture in FFPE tissue sections. Captured DNA is then used for Sanger or gene-targeted deep sequencing to detect known and/or novel drivers of aldosterone production.

**Figure 2:**
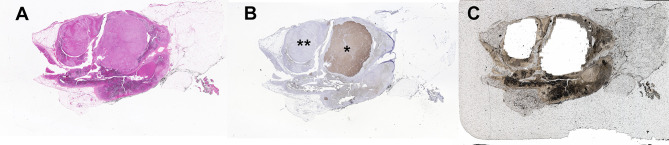
Example of a multinodular adrenal sample from a patient with primary aldosteronism that illustrates the benefit of CYP11B2 IHC-guided DNA capture. **(A)** Hematoxylin and eosin staining, **(B)** CYP11B2 immunohistochemistry; *, aldosterone-producing adenoma (APA); **, CYP11B2-negative tumor, **(C)** Post-captured unstained FFPE adrenal tissue section. For DNA isolation, an APA and a CYP11B2-negative tumor were selectively scraped based on the results of CYP11B2 immunohistochemistry.

Traditionally, DNA and RNA have been isolated from snap frozen tumor pieces obtained during pathologic gross dissection at the time of adrenalectomy. Mutational analysis has subsequently been performed without consideration of CYP11B2 expression prior to sequencing. In the largest mutation prevalence study using this conventional approach, aldosterone-driver somatic mutations were detected in 54% of 474 adrenal tumors from PA patients (55). Considering the aforementioned diversities in the histology of PA, this approach could negatively affect the accuracy of mutational analysis. As such, we recently developed an advanced molecular profiling method using selective DNA isolation from FFPE sections based on CYP11B2 IHC, followed by NGS (14, 56). The step-by-step sequencing method using the CYP11B2 IHC-guided approach is shown in **Figure 1**. Many laboratories, including ours, use a mouse monoclonal antibody specific for human CYP11B2 that was produced and characterized by Dr. Celso Gomez-Sanchez (46). This antibody is commercially available from Millipore Sigma (MABS1251, RRID: AB_2783793), making it useful for both research and pathologic diagnosis purposes. As is needed for most antibodies, laboratory testing for individual in-house protocols should be done to optimize specificity and sensitivity for CYP11B2 detection. Initial protocol testing is particularly important due to the variable CYP11B2 expression seen between APAs. The scanned slide images of adrenal tumor tissue from a PA patient are shown in **Figure 2**. The adrenal contains two distinct adrenocortical tumors (an APA and a CYP11B2-negative tumor) which exist close to each other. This example highlights the importance of targeted DNA capture method for accurate mutation analysis. Importantly, past studies demonstrated that no aldosterone-driver mutation was detected in CYP11B2-negative adrenocortical tumors from PA patients (49, 57).

Using this CYP11B2 IHC-guided approach, aldosterone-driver mutations have been identified in 88-96% of APAs (14, 48, 49). A recent study demonstrated a better mutation detection rate using CYP11B2-IHC guided sequencing (94%) as compared to the authors’ previous use of conventional tumor tissue approaches (71%) (58).

**Figure 3:**
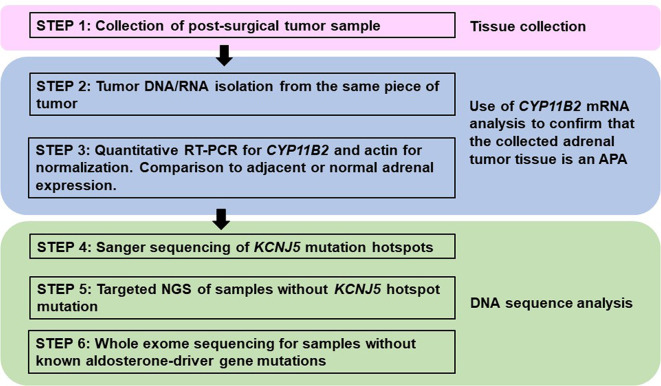
Proposed method for processing fresh or frozen adrenal tumor tissue from patients with primary aldosteronism for mutational analysis. This approach varies from FFPE processing (Figure 1) by the method of tissue collection and the need to use *CYP11B2* mRNA detection for confirmation of APA status. However, mutational analysis is similar using Sanger and/or next-generation sequencing to detect known and/or novel drivers of aldosterone production.

For the laboratories using traditional material, i.e., DNA/RNA from macro-dissected snap frozen tumor pieces, confirmation of *CYP11B2* mRNA expression by quantitative reverse transcription polymerase chain reaction (RT-qPCR) prior to sequencing could also improve the mutation detection rate. A proposed method for mutational analysis using banked snap frozen material is shown in **Figure 3**.

## Defining Somatic Mutations in Aldosterone-Producing Lesions

### Sanger Sequencing

Traditional direct Sanger sequencing has been widely used for the mutational analysis of APA. As new APA-related genes have continuously been identified, it is challenging to perform Sanger sequencing for the screening of multiple genes – particularly for genes like *CACNA1D*, which have a large coding region with dispersed mutation hotspot areas. Targeted NGS is rapidly becoming the preferred method due to its high sensitivity and ability to utilize small amounts of DNA; however, Sanger sequencing is still an attractive method considering the high per sample cost of NGS. As the prevalence of *KCNJ5* hotspot mutations in APA is relatively high, one option to decrease sample throughput is screening for *KCNJ5* mutation hotspots using Sanger sequencing, followed by targeted NGS of *KCNJ5* mutation-negative samples (29, 48) (**Figure 1**). This approach significantly reduces cost and can also be applied to material isolated from traditional snap frozen tissue (**Figure 3**). For researchers without available NGS to screen entire coding regions, targeted Sanger sequencing that covers the majority of known aldosterone-driver mutations can be done in a systematic manner. Based on the APA mutation prevalence from our previous study (14), the use of five primer pairs (one for the *KCNJ5*, one for *ATP1A1*, one for *ATP2B3*, and two for *CACNA1D*, **Table 2**) appear to be able to identify over 70% of mutations by direct Sanger sequencing in a Caucasian American cohort. Special consideration is required for primer design when using genomic DNA (gDNA) from FFPE as a template, since FFPE-extracted DNA can be heavily degraded and fragmented. The authors recommend designing primer sets that target the amplicon size below 250 base pairs (bp) if possible.

**Table 2:** PCR primer sets for aldosterone-driver mutation hotspots.

Gene	Exon	Primer Sequences		Amplicon Size (bp)	Reference
*KCNJ5*	2	Forward	GGACCATGTTGGCGACCAAGAGTG	211	(21)
		Reverse	GACAAACATGCACCCCACCATGAAG		
*ATP1A1*	4	Forward	ATTAACATCTGCTCGTGCAGCTGAG	227	
		Reverse	CCATATGCTGAATTACAGAACTCAC		
*ATP2B3*	8	Forward	TGTCTGCCATCACCGTCATCATC	255	(14)
		Reverse	CCCAGTTTCCGAGTCTGTAAACAG		
*CACNA1D*	8A	Forward	CCCACTCCTATGAGACCATC	190	
		Reverse	TCTTGGCAACTGTCCTCAGG		
	16	Forward	GGTGTGTGGCGTTGCCATTG	253	(29)
		Reverse	AACTGTTGCAGGGCTCCCA		

### Next-Generation Sequencing

NGS has rapidly become the standard approach for comprehensive molecular profiling of human tumors due to its ability to generate sequence-level genetic data simultaneously for tens, hundreds, or even thousands of genes. Although a variety of NGS methods and platforms exist, there are two broad approaches: amplicon-based and hybridization capture-based (**Table 3**). Amplicon-based approaches utilize multiplex PCR reactions to amplify genomic regions of interest, while hybridization capture-based methods utilize biotinylated oligonucleotide baits to pull down target regions from pools of sheared gDNA. In general, amplicon-based methods are preferred for targeted sequencing of small numbers of genomic regions or when available input DNA for NGS library preparation is very low – particularly for FFPE samples – while hybridization capture-based approaches are favored for analyzing a large number of genomic regions [e.g., whole-exome sequencing (WES)] when ample input DNA is available. These and other differences between the NGS approaches inform how they may be best utilized for molecular profiling of aldosterone-producing lesions using FFPE tissue (**Figure 1**).

**Table 3:** Comparison of NGS approaches for molecular profiling of aldosterone-producing adrenal cortical lesions.

	Amplicon-based	Hybridization Capture-based
Enrichment method	Multiplex PCR	Biotinylated oligonucleotide baits
Input DNA	Less	More
# of genomic targets	Fewer	More
Experimental time	Less	More
Cost per sample	Lower*	Higher*
Application(s)	Targeted sequencing	Targeted sequencing or WES

*Depends on depth of sequencing and # of genomic targets.

WES, whole-exome sequencing.

Given the relatively limited number of established aldosterone-driver mutations – coupled with the fact that most of these mutations occur at specific hotspot regions within the affected genes – targeted amplicon-based NGS is ideal for characterizing FFPE APA samples. As mentioned earlier, recent studies utilizing this approach have identified somatic aldosterone-driver mutations in the vast majority of APA. In addition to the ability to interrogate multiple genomic regions simultaneously, one of the important advantages of NGS over Sanger sequencing is improved sensitivity for detecting genetic variants. This is particularly important for detecting somatic mutations in microscopic lesions (i.e., APCC/APM), for which the expected allelic variant fraction may be less than 20% (depending on the purity of the isolated tissue for sequencing). Application of targeted amplicon-based NGS to APCC in normal adrenal glands and from patients with adrenal idiopathic hyperaldosteronism has identified somatic aldosterone-driver mutations in 34-58% of these lesions (50–52). For aldosterone-producing lesions that are mutation-negative by targeted amplicon-based NGS, hybridization capture-based WES of CYP11B2 IHC-guided FFPE tissue may identify novel aldosterone-driver mutations (9, 36). Finally, despite several clear advantages of NGS-based molecular profiling, application of these approaches to FFPE tissue is potentially limited by FFPE-associated DNA degradation (e.g., increased genomic fragmentation, artifactual nucleotide deamination) and technical issues (e.g., PCR amplification bias, sequencing error). Emerging NGS methods, including the use of unique molecular identifiers (UMI; as known as “molecular barcodes”), and novel NGS technologies may begin to address some of these limitations and will continue to revolutionize genomic characterization of human tumors, including aldosterone-producing lesions.

## Conclusions

Recent advances in sequencing technology have significantly accelerated PA research to elucidate its molecular pathogenesis. Unique histologic characteristics of adrenals from patients with PA require special attention to tumor CYP11B2 expression for accurate somatic mutation identification. The streamlined approach using CYP11B2 IHC-guided DNA capture combined with NGS appears to be a preferred method for mutational analysis of adrenals from patients with PA. The use of this CYP11B2 IHC-guided sequencing approach in a large prospective cohort will allow us to accurately determine APA mutation prevalence as well as genotype-phenotype correlations.

## Author Contributions

KN and WR conceived the idea of this review article. KN and AU drafted the manuscript. WR reviewed the manuscript and made edits on the contents. All authors contributed to the article and approved the submitted version.

## Funding

This work was supported by grants from National Institutes of Diabetes and Digestive and Kidney Disease (DK106618 and DK043140) to WR. KN is supported by the Japan Heart Foundation Research Grant.

## Conflict of Interest

The authors declare that the research was conducted in the absence of any commercial or financial relationships that could be construed as a potential conflict of interest.

## References

[1] Choi M Scholl UI Yue P Bjorklund P Zhao B Nelson-Williams C. K+ Channel Mutations in Adrenal Aldosterone-Producing Adenomas and Hereditary Hypertension. Science (2011) 331:768–72. 10.1126/science.1198785 PMC3371087 21311022

[2] Beuschlein F Boulkroun S Osswald A Wieland T Nielsen HN Lichtenauer UD. Somatic Mutations in ATP1A1 and ATP2B3 Lead to Aldosterone-Producing Adenomas and Secondary Hypertension. Nat Genet (2013) 45:440–4, 444e1-2. 10.1038/ng.2550 23416519

[3] Scholl UI Goh G Stolting G de Oliveira RC Choi M Overton JD. Somatic and Germline CACNA1D Calcium Channel Mutations in Aldosterone-Producing Adenomas and Primary Aldosteronism. Nat Genet (2013) 45:1050–4. 10.1038/ng.2695 PMC3876926 23913001

[4] Azizan EA Poulsen H Tuluc P Zhou J Clausen MV Lieb A. Somatic Mutations in ATP1A1 and CACNA1D Underlie a Common Subtype of Adrenal Hypertension. Nat Genet (2013) 45:1055–60. 10.1038/ng.2716 23913004

[5] Scholl UI Stolting G Nelson-Williams C Vichot AA Choi M Loring E. Recurrent Gain of Function Mutation in Calcium Channel CACNA1H Causes Early-Onset Hypertension With Primary Aldosteronism. Elife (2015) 4:e06315. 10.7554/eLife.06315 25907736 PMC4408447

[6] Scholl UI Stolting G Schewe J Thiel A Tan H Nelson-Williams C. CLCN2 Chloride Channel Mutations in Familial Hyperaldosteronism Type II. Nat Genet (2018) 50:349–54. 10.1038/s41588-018-0048-5 PMC5862758 29403011

[7] Fernandes-Rosa FL Daniil G Orozco IJ Goppner C El Zein R Jain V. A Gain-of-Function Mutation in the CLCN2 Chloride Channel Gene Causes Primary Aldosteronism. Nat Genet (2018) 50:355–61. 10.1038/s41588-018-0053-8 29403012

[8] Dutta RK Arnesen T Heie A Walz M Alesina P Soderkvist P. A Somatic Mutation in CLCN2 Identified in a Sporadic Aldosterone-Producing Adenoma. Eur J Endocrinol (2019) 181:K37–41. 10.1530/EJE-19-0377 31491746

[9] Nanba K Blinder AR Rege J Hattangady NG Else T Liu CJ. Somatic CACNA1H Mutation As a Cause of Aldosterone-Producing Adenoma. Hypertension (2020) 75:645–9. 10.1161/HYPERTENSIONAHA.119.14349 PMC7059016 31983310

[10] Fernandes-Rosa FL Boulkroun S Zennaro MC. Genetic and Genomic Mechanisms of Primary Aldosteronism. Trends Mol Med (2020) 26:819–32. 10.1016/j.molmed.2020.05.005 32563556

[11] Akerstrom T Maharjan R Sven Willenberg H Cupisti K Ip J Moser A. Activating Mutations in CTNNB1 in Aldosterone Producing Adenomas. Sci Rep (2016) 6:19546. 10.1038/srep19546 26815163 PMC4728393

[12] Wu VC Wang SM Chueh SJ Yang SY Huang KH Lin YH. The Prevalence of CTNNB1 Mutations in Primary Aldosteronism and Consequences for Clinical Outcomes. Sci Rep (2017) 7:39121. 10.1038/srep39121 28102204 PMC5244399

[13] Scholl UI Healy JM Thiel A Fonseca AL Brown TC Kunstman JW. Novel Somatic Mutations in Primary Hyperaldosteronism Are Related to the Clinical, Radiological and Pathological Phenotype. Clin Endocrinol (Oxf) (2015) 83:779–89. 10.1111/cen.12873 PMC4995792 26252618

[14] Nanba K Omata K Else T Beck PCC Nanba AT Turcu AF. Targeted Molecular Characterization of Aldosterone-Producing Adenomas in White Americans. J Clin Endocrinol Metab (2018) 103:3869–76. 10.1210/jc.2018-01004 PMC6179168 30085035

[15] Cheng CJ Sung CC Wu ST Lin YC Sytwu HK Huang CL. Novel KCNJ5 Mutations in Sporadic Aldosterone-Producing Adenoma Reduce Kir3.4 Membrane Abundance. J Clin Endocrinol Metab (2015) 100(1):E155–63. 10.1210/jc.2014-3009 25347571

[16] Williams TA Monticone S Schack VR Stindl J Burrello J Buffolo F. Somatic ATP1A1, ATP2B3, and KCNJ5 Mutations in Aldosterone-Producing Adenomas. Hypertension (2014) 63(1):188–95. 10.1161/HYPERTENSIONAHA.113.01733 24082052

[17] Hardege I Xu S Gordon RD Thompson AJ Figg N Stowasser M. Novel Insertion Mutation in KCNJ5 Channel Produces Constitutive Aldosterone Release From H295R Cell. Mol Endocrinol (2015) 29(10):1522–30. 10.1210/me.2015-1195 PMC5414677 26340408

[18] Akerstrom T Crona J Delgado Verdugo A Starker LF Cupisti K Willenberg HS. Comprehensive Re-Sequencing of Adrenal Aldosterone Producing Lesions Reveal Three Somatic Mutations Near the KCNJ5 Potassium Channel Selectivity Filter. PloS One (2012) 7(7):e41926. 22848660 10.1371/journal.pone.0041926 PMC3407065

[19] Zheng FF Zhu LM Zhou WL Zhang Y Li MY Zhu YC. A Novel Somatic Mutation 145-147deleteinsk in KCNJ5 Increases Aldosterone Production. J Hum Hypertens (2017) 31(11):756–9. 10.1038/jhh.2017.50 28974779

[20] Wang B Li X Zhang X Ma X Chen L Zhang Y. Prevalence and Characterization of Somatic Mutations in Chinese Aldosterone-Producing Adenoma Patients. Med (Baltimore) (2015) 94(16):e708. 10.1097/MD.0000000000000708 PMC4602684 25906099

[21] Nanba K Omata K Tomlins SA Giordano TJ Hammer GD Rainey WE. Double Adrenocortical Adenomas Harboring Independent KCNJ5 and PRKACA Somatic Mutations. Eur J Endocrinol (2016) 175(2):K1–6. 10.1530/EJE-16-0262 PMC5030510 27165862

[22] Zheng FF Zhu LM Nie AF Li XY Lin JR Zhang K. Clinical Characteristics of Somatic Mutations in Chinese Patients With Aldosterone-Producing Adenoma. Hypertension (2015) 65(3):622–8. 10.1161/HYPERTENSIONAHA.114.03346 25624344

[23] Kuppusamy M Caroccia B Stindl J Bandulik S Lenzini L Gioco F. A Novel KCNJ5-Inst149 Somatic Mutation Close to, But Outside, the Selectivity Filter Causes Resistant Hypertension by Loss of Selectivity for Potassium. J Clin Endocrinol Metab (2014) 99(9):E1765–73. 10.1210/jc.2014-1927 PMC4154085 25057880

[24] Taguchi R Yamada M Nakajima Y Satoh T Hashimoto K Shibusawa N. Expression and Mutations of KCNJ5 mRNA in Japanese Patients With Aldosterone-Producing Adenomas. J Clin Endocrinol Metab (2012) 97(4):1311–9. 10.1210/jc.2011-2885 22278422

[25] Kitamoto T Omura M Suematsu S Saito J Nishikawa T. KCNJ5 Mutation as a Predictor for Resolution of Hypertension After Surgical Treatment of Aldosterone-Producing Adenoma. J Hypertens (2018) 36(3):619–27. 10.1097/HJH.0000000000001578 29016532

[26] Azizan EA Lam BY Newhouse SJ Zhou J Kuc RE Clarke J. Microarray, qPCR, and KCNJ5 Sequencing of Aldosterone-Producing Adenomas Reveal Differences in Genotype and Phenotype Between Zona Glomerulosa- and Zona Fasciculata-Like Tumors. J Clin Endocrinol Metab (2012) 97(5):E819–29. 10.1210/jc.2011-2965 22442279

[27] Mulatero P Tauber P Zennaro MC Monticone S Lang K Beuschlein F. KCNJ5 Mutations in European Families With Nonglucocorticoid Remediable Familial Hyperaldosteronism. Hypertension (2012) 59(2):235–40. 10.1161/HYPERTENSIONAHA.111.183996 22203740

[28] Akerstrom T Willenberg HS Cupisti K Ip J Backman S Moser A. Novel Somatic Mutations and Distinct Molecular Signature in Aldosterone-Producing Adenomas. Endocr Relat Cancer (2015) 22(5):735–44. 10.1530/ERC-15-0321 26285814

[29] Guo Z Nanba K Udager A McWhinney BC Ungerer JPJ Wolley M. Biochemical, Histopathological, and Genetic Characterization of Posture-Responsive and Unresponsive APA. J Clin Endocrinol Metab (2020) 105(9):e3224–35. 10.1210/clinem/dgaa367 PMC7426003 32516371

[30] Backman S Akerstrom T Maharjan R Cupisti K Willenberg HS Hellman P. RNA Sequencing Provides Novel Insights Into the Transcriptome of Aldosterone Producing Adenoma. Sci Rep (2019) 9(1):6269. 31000732 10.1038/s41598-019-41525-2 PMC6472367

[31] Wu VC Huang KH Peng KY Tsai YC Wu CH Wang SM. Prevalence and Clinical Correlates of Somatic Mutation in Aldosterone Producing Adenoma-Taiwanese Population. Sci Rep (2015) 5:11396. 26066391 10.1038/srep11396 PMC4464349

[32] Murakami M Yoshimoto T Minami I Bouchi R Tsuchiya K Hashimoto K. A Novel Somatic Deletion Mutation of ATP2B3 in Aldosterone-Producing Adenom. Endocr Pathol (2015) 26(4):328–33. 10.1007/s12022-015-9400-9 26481629

[33] Kitamoto T Suematsu S Yamazaki Y Nakamura Y Sasano H Matsuzawa Y. Clinical and Steroidogenic Characteristics of Aldosterone-Producing Adenomas With ATPase or CACNA1D Gene Mutation. J Clin Endocrinol Metab (2016) 101(2):494–503. 26606680 10.1210/jc.2015-3284

[34] Dutta RK Welander J Brauckhoff M Walz M Alesina P Arnesen T. Complementary Somatic Mutations of KCNJ5, ATP1A1, and ATP2B3 in Sporadic Aldosterone Producing Adrenal Adenomas. Endocr Relat Cancer (2014) 21(1):L1–4. 10.1530/ERC-13-0466 24179102

[35] Tan GC Negro G Pinggera A Tizen Laim NMS Mohamed Rose I Ceral J. Aldosterone-Producing Adenomas: Histopathology-Genotype Correlation and Identification of a Novel CACNA1D Mutatio. Hypertension (2017) 70(1):129–36. 10.1161/HYPERTENSIONAHA.117.09057 28584016

[36] Rege J Nanba K Blinder AR Plaska S Udager AM Vats P. Identification of Somatic Mutations in CLCN2 in Aldosterone-Producing Adenomas. J Endocr Soc (2020) 4:bvaa123. 10.1210/jendso/bvaa123 33033789 PMC7528565

[37] Kem DC Weinberger MH Higgins JR Kramer NJ Gomez-Sanchez C Holland OB. Plasma Aldosterone Response to ACTH in Primary Aldosteronism and in Patients With Low Renin Hypertension. J Clin Endocrinol Metab (1978) 46:552–60. 10.1210/jcem-46-4-552 225341

[38] Lefebvre H Cartier D Duparc C Lihrmann I Contesse V Delarue C. Characterization of Serotonin(4) Receptors in Adrenocortical Aldosterone-Producing Adenomas: *In Vivo* and *In Vitro* Studies. J Clin Endocrinol Metab (2002) 87:1211–6. 10.1210/jcem.87.3.8327 11889190

[39] Zwermann O Suttmann Y Bidlingmaier M Beuschlein F Reincke M. Screening for Membrane Hormone Receptor Expression in Primary Aldosteronism. Eur J Endocrinol (2009) 160:443–51. 10.1530/EJE-08-0711 19131502

[40] Albiger NM Sartorato P Mariniello B Iacobone M Finco I Fassina A. A Case of Primary Aldosteronism in Pregnancy: Do LH and GNRH Receptors Have a Potential Role in Regulating Aldosterone Secretion? Eur J Endocrinol (2011) 164:405–12. 10.1530/EJE-10-0879 21330483

[41] Lopez AG Duparc C Naccache A Castanet M Lefebvre H Louiset E. Role of Mast Cells in the Control of Aldosterone Secretion. Horm Metab Res (2020) 52:412–20. 10.1055/a-1119-1063 32215882

[42] Sonoyama T Sone M Miyashita K Tamura N Yamahara K Park K. Significance of Adrenocorticotropin Stimulation Test in the Diagnosis of an Aldosterone-Producing Adenoma. J Clin Endocrinol Metab (2011) 96:2771–8. 10.1210/jc.2011-0573 21752891

[43] El Ghorayeb N Bourdeau I Lacroix A. Role of ACTH and Other Hormones in the Regulation of Aldosterone Production in Primary Aldosteronism. Front Endocrinol (Lausanne) (2016) 7:72. 10.3389/fendo.2016.00072 27445975 PMC4921457

[44] St-Jean M Bourdeau I Martin M Lacroix A. Aldosterone is Aberrantly Regulated by Various Stimuli in a High Proportion of Patients With Primary Aldosteronism. J Clin Endocrinol Metab (2021) 106:e45–60. 10.1210/clinem/dgaa703 PMC7765652 33000146

[45] Nishimoto K Nakagawa K Li D Kosaka T Oya M Mikami S. Adrenocortical Zonation in Humans Under Normal and Pathological Conditions. J Clin Endocrinol Metab (2010) 95:2296–305. 10.1210/jc.2009-2010 20200334

[46] Gomez-Sanchez CE Qi X Velarde-Miranda C Plonczynski MW Parker CR Rainey W. Development of Monoclonal Antibodies Against Human CYP11B1 and CYP11B2. Mol Cell Endocrinol (2014) 383:111–7. 10.1016/j.mce.2013.11.022 PMC3939805 24325867

[47] Williams TA Gomez-Sanchez CE Rainey WE Giordano TJ Lam AK Marker A. International Histopathology Consensus for Unilateral Primary Aldosteronism. J Clin Endocrinol Metab (2021) 106:42–54. 10.1210/clinem/dgaa484 32717746 PMC7765663

[48] Nanba K Yamazaki Y Bick N Onodera K Tezuka Y Omata K. Prevalence of Somatic Mutations in Aldosterone-Producing Adenomas in Japanese Patients. J Clin Endocrinol Metab (2020) 105:e4066–73. 10.1210/clinem/dgaa595 PMC7947976 32844168

[49] Nanba K Omata K Gomez-Sanchez CE Stratakis CA Demidowich AP Suzuki M. Genetic Characteristics of Aldosterone-Producing Adenomas in Blacks. Hypertension (2019) 73:885–92. 10.1161/HYPERTENSIONAHA.118.12070 PMC6416065 30739536

[50] Nishimoto K Tomlins SA Kuick R Cani AK Giordano TJ Hovelson DH. Aldosterone-Stimulating Somatic Gene Mutations Are Common in Normal Adrenal Glands. Proc Natl Acad Sci USA (2015) 112:E4591–9. 10.1073/pnas.1505529112 PMC4547250 26240369

[51] Omata K Anand SK Hovelson DH Liu CJ Yamazaki Y Nakamura Y. Aldosterone-Producing Cell Clusters Frequently Harbor Somatic Mutations and Accumulate With Age in Normal Adrenals. J Endocr Soc (2017) 1:787–99. 10.1210/js.2017-00134 PMC5686701 29264530

[52] Omata K Satoh F Morimoto R Ito S Yamazaki Y Nakamura Y. Cellular and Genetic Causes of Idiopathic Hyperaldosteronism. Hypertension (2018) 72:874–80. 10.1161/HYPERTENSIONAHA.118.11086 PMC6207209 30354720

[53] Nanba AT Nanba K Byrd JB Shields JJ Giordano TJ Miller BS. Discordance Between Imaging and Immunohistochemistry in Unilateral Primary Aldosteronism. Clin Endocrinol (Oxf) (2017) 87:665–72. 10.1111/cen.13442 PMC5698145 28787766

[54] Fernandes-Rosa FL Giscos-Douriez I Amar L Gomez-Sanchez CE Meatchi T Boulkroun S. Different Somatic Mutations in Multinodular Adrenals With Aldosterone-Producing Adenoma. Hypertension (2015) 66:1014–22. 10.1161/HYPERTENSIONAHA.115.05993 PMC4600038 26351028

[55] Fernandes-Rosa FL Williams TA Riester A Steichen O Beuschlein F Boulkroun S. Genetic Spectrum and Clinical Correlates of Somatic Mutations in Aldosterone-Producing Adenoma. Hypertension (2014) 64:354–61. 10.1161/HYPERTENSIONAHA.114.03419 24866132

[56] Nanba K Chen AX Omata K Vinco M Giordano TJ Else T. Molecular Heterogeneity in Aldosterone-Producing Adenomas. J Clin Endocrinol Metab (2016) 101:999–1007. 10.1210/jc.2015-3239 26765578 PMC4803171

[57] Dekkers T ter Meer M Lenders JW Hermus AR Schultze Kool L Langenhuijsen JF. Adrenal Nodularity and Somatic Mutations in Primary Aldosteronism: One Node is the Culprit? J Clin Endocrinol Metab (2014) 99:E1341–51. 10.1210/jc.2013-4255 24758183

[58] De Sousa K Boulkroun S Baron S Nanba K Wack M Rainey WE. Genetic, Cellular, and Molecular Heterogeneity in Adrenals With Aldosterone-Producing Adenoma. Hypertension (2020) 75:1034–44. 10.1161/HYPERTENSIONAHA.119.14177 PMC7098445 32114847

